# Characterization of ESBL-Producing *Escherichia coli* and *Klebsiella pneumoniae* Isolated from Clinical Samples in a Northern Portuguese Hospital: Predominance of CTX-M-15 and High Genetic Diversity

**DOI:** 10.3390/microorganisms9091914

**Published:** 2021-09-09

**Authors:** Isabel Carvalho, José António Carvalho, Sandra Martínez-Álvarez, Madjid Sadi, Rosa Capita, Carlos Alonso-Calleja, Fazle Rabbi, Maria de Lurdes Nunes Enes Dapkevicius, Gilberto Igrejas, Carmen Torres, Patrícia Poeta

**Affiliations:** 1 Microbiology and Antibiotic Resistance Team (MicroART), Department of Veterinary Sciences, University of Trás-os-Montes and Alto Douro, 5000-801 Vila Real, Portugal; isabelbarrosocarvalho@utad.pt; 2 Department of Genetics and Biotechnology, University of Trás-os-Montes and Alto Douro, 5000-801 Vila Real, Portugal; gigrejas@utad.pt; 3 Functional Genomics and Proteomics Unit, University of Trás-os-Montes and Alto Douro, 5000-801 Vila Real, Portugal; 4 Laboratory Associated for Green Chemistry (LAQV-REQUIMTE), New University of Lisbon, 2829-516 Monte da Caparica, Portugal; 5 Area Biochemistry and Molecular Biology, University of La Rioja, 26006 Logroño, Spain; sandra.martinezal@alum.unirioja.es (S.M.-Á.); sadimadjid@gmail.com (M.S.); carmen.torres@unirioja.es (C.T.); 6 Medical Center of Trás-os-Montes e Alto Douro E.P.E., 5000-508 Vila Real, Portugal; j.m.c.carvalho@sapo.pt; 7 Laboratory of Biotechnology Related to Animals Reproduction, Université Saad Dahlab de Blida, Blida 09000, Algeria; 8 Department of Food Hygiene and Technology, Veterinary Faculty, University of León, 24071 León, Spain; rosa.capita@unileon.es (R.C.); carlos.alonso.calleja@unileon.es (C.A.-C.); 9 Institute of Food Science and Technology, University of León, 24071 León, Spain; 10 Australian Computer Society, Melbourne 3008, Australia; bilirabbi@yahoo.com; 11 Faculty of Agricultural and Environmental Sciences, University of the Azores, 9500-321 Angra do Heroísmo, Portugal; maria.ln.dapkevicius@uac.pt; 12 Institute of Agricultural and Environmental Research and Technology (IITAA), University of the Azores, 9500-321 Azores, Portugal

**Keywords:** antimicrobial resistance, *Klebsiella pneumoniae*, *Escherichia coli*, public health, Carbapenemases, β-lactamases, KPC2/3, CTX-M-15, human, Portugal

## Abstract

Background: *Enterobacteriaceae* are major players in the spread of resistance to β-lactam antibiotics through the action of CTX-M β-lactamases. We aimed to analyze the diversity and genetic characteristics of ESBL-producing *Escherichia coli* and *Klebsiella pneumoniae* isolates from patients in a Northern Portuguese hospital. Methods: A total of 62 cefotaxime/ceftazidime-resistant *E. coli* (*n* = 38) and *K. pneumoniae* (*n* = 24) clinical isolates were studied. Identification was performed by MALDI-TOF MS. Antimicrobial susceptibility testing against 13 antibiotics was performed. Detection of ESBL-encoding genes and other resistance genes, phylogenetic grouping, and molecular typing (for selected isolates) was carried out by PCR/sequencing. Results: ESBL activity was detected in all 62 *E. coli* and *K. pneumoniae* isolates. Most of the ESBL-producing *E. coli* isolates carried a *bla*_CTX-M_ gene (37/38 isolates), being *bla*_CTX-M-15_ predominant (*n* = 32), although *bla*_CTX-M-27_ (*n* = 1) and *bla*_CTX-M-1_ (*n* = 1) were also detected. Two *E. coli* isolates carried the *bla*_KPC2/3_ gene. The lineages ST131-B2 and ST410-A were detected among the ESBL-producing blood *E. coli* isolates. Regarding the 24 ESBL-producing *K. pneumoniae* isolates, 18 carried a *bla*_CTX-M_ gene (*bla*_CTX-M-15_, 16 isolates; *bla*_CTX-M-55_, 2 isolates). All *K. pneumoniae* isolates carried *bla*_SHV_ genes, including ESBL-variants (*bla*_SHV-12_ and *bla*_SHV-27_, 14 isolates) or non-ESBL-variants (*bla*_SHV-11_ and *bla*_SHV-28_, 10 isolates); ten *K. pneumoniae* isolates also carried the *bla*_KPC2/3_ gene and showed imipenem-resistance. ESBL-positive *E. coli* isolates were ascribed to the B_2_ phylogenetic group (82%), mostly associated with ST131 lineage and, at a lower rate, to ST410/A. Regarding *K. pneumoniae*, the three international lineages ST15, ST147, and ST280 were detected among selected isolates. Conclusions: Different ESBL variants of CTX-M (especially CTX-M-15) and SHV-type (specially SHV-12) were detected among CTX/CAZ^R^ *E. coli* and *K. pneumoniae* isolates, in occasions associated with carbapenemase genes (*bla*_KPC2/3_ gene).

## 1. Introduction

*Escherichia coli* is a commensal microorganism of the intestinal microbiota of humans and animals. It is also involved in a great variety of intestinal and extra-intestinal infections as an opportunistic pathogen, including septicemia, as well as urinary and wound infections, among others; this microorganism presents a high facility to acquire antimicrobial-resistant genes [1,2]. In addition, *Klebsiella pneumoniae* can be found in the intestinal microbiota of healthy humans and animals although may also cause life-threatening infections; it is considered a major opportunistic pathogen implicated in nosocomial infections (as is the case of pneumonia or bloodstream infections), especially in patients of intensive-care units (UCIs) and in immunocompromised hosts with severe underlying diseases. *K. pneumoniae* strains can accumulate resistance genes, increasing their pathogenicity and causing severe infections [1,3,4,5]. Moreover, it is known that *K. pneumoniae* spreads easily, mainly in the hospital environment [5].

During recent years, the emergence and rapid dissemination of *Enterobacteriaceae* carrying genes encoding Extended Spectrum β-lactamases (ESBL), acquired AmpC β-lactamases (qAmpC), or carbapenemases are considered great concerns [6]. In particular, ESBLs of CTX-M-type and qAmpC enzymes (especially the CMY-2 type) were increasingly reported worldwide, specifically among clinical isolates from *E. coli* [7,8,9] and *K. pneumoniae* [6,10,11,12]. Moreover, recent studies reported ESBL-producing *K. pneumoniae* isolates among urinary tract infections and invasive infections in patients in Portugal [4,5,9], as well as among healthy Portuguese students in Lisbon [13]. Furthermore, a previous report created by our group showed the presence of ESBL-producing *E. coli* among healthy and sick cats [14] and dogs [15] in Portugal.

According to a recent review conducted by Sengodan et al. [16], more than eighty CTX-M variants have been reported worldwide; the majority of them are more active on cefotaxime than on ceftazidime. Particularly, the CTX-M-15 is frequently found among *Enterobacteriaceae* in humans in Europe [17,18], specifically in both clinical samples and healthy humans in Portugal [19,20,21]. Moreover, the CTX-M-15 β-lactamase is frequently associated with the uropathogenic international *E. coli* clone ST131 [22,23]. In the last decade, *E. coli* clonal group ST131 has emerged as a high-risk clone with important clinical health concerns causing multidrug-resistant (MDR) infections worldwide [11]. Particularly in Portuguese territory, ST131 clone was detected among residents of nursing homes [22], sick dogs [15], as well as in UTI clinical isolates from humans [24]. Nevertheless, other high-risk international clones have been detected among ESBL- or carbapenemase-producing *E. coli* isolates, as is the case of ST410 [25,26,27]. Other high-risk *E. coli* clones have been extensively reported in extraintestinal human infections [28].

According to Domokos et al. [18], high-risk *K. pneumoniae* clones, such as ST11 and ST15, have the tenacity and flexibility to accumulate resistance determinants, contributing to the increase of their pathogenicity. Regarding a recent study, the ST258, ST11, ST15, and ST147 clones spread for two decades, and recently, the CTX-M-15-producing ST307 clone in *K. pneumoniae* emerged globally [29]. Particularly, carbapenems are considered last-resort treatment but *K. pneumoniae* acquired resistance to this last resort antibiotic worldwide [30].

On the other hand, a great diversity of SHV variants have been reported, being a group of them of ESBL-type [31] being frequently detected among *K. pneumoniae* isolates, especially the SHV-5 and SHV-12 variants [1,16].

Antimicrobial resistance is commonly related to the spread of plasmids and the acquisition of resistance genes that normally occur by horizontal gene transfer (HGT). Another important aspect is the mutational events, mainly in sequences of genes encoding the target for certain antibiotics. Another mechanism of resistance (mainly in quinolones) is the alteration of the outer membrane proteins associated with active efflux pumps, which finish with the expulsion of the antibiotic out of the bacterial cell [1,16].

To our knowledge, some studies have been performed in Portugal based on the detection of ESBL-producing *Enterobacteriaceae* in hospitalized patients [3,4,12,19,32,33,34,35,36]. In a previous study performed by our research group, the ESBLs types were determined among invasive *K. pneumoniae* isolates recovered from blood cultures in a hospital located in Northern Portugal [5]. The purpose of the present study is to expand this previous work by analyzing in the same hospital the ESBLs types and the main associated resistance mechanisms in broad-spectrum cephalosporin-resistant *K. pneumoniae* isolates obtained from different clinical origins (except blood), and also in *Escherichia coli* isolates obtained from blood and urine samples; furthermore, the genetic lineages of selected isolates were also the aim of this research.

## 2. Materials and Methods

### 2.1. Bacterial Isolates

A collection of 38 cefotaxime/ceftazidime-resistant (CTX/CAZ^R^) *E. coli* isolates (18 of urine samples and 20 from blood samples) were obtained from hospitalized patients (one isolate/patient) in a Northern Portuguese hospital (Centro Hospitalar de Trás os Montes e Alto Douro, CHTMAD, Vila Real), between December 2016 and August 2018. A collection of 24 CTX/CAZ^R^ *K. pneumoniae* isolates (18 from the urine; three of bronchial secretion and three from pus/biopsy/catheter origins) were also collected from the same hospital between December 2016 and December 2017. The identification of the isolates was confirmed by the matrix-assisted laser desorption-ionization time-of-flight mass spectrometry method (MALDI-TOF MS) [37,38], following the instructions of the manufacturer (Bruker Daltonik, Bremen, Germany).

### 2.2. Antimicrobial Susceptibility Testing

Antimicrobial susceptibility testing was performed by using Kirby–Bauer disk diffusion method on Mueller-Hinton agar, according to Clinical Laboratory Standards Institute guidelines (CLSI, 2019) [39]. The susceptibility of *E. coli* and *K. pneumoniae* isolates was tested for the following antibiotics (μg/disk): amoxicillin + clavulanic acid (20 + 10), cefoxitin (30), ceftazidime (30), cefotaxime (30), imipenem (10), tetracycline (30), gentamicin (10), streptomycin (10), tobramycin (10), ciprofloxacin (5) and trimethoprim-sulfamethoxazole (1.25 + 23.75). Moreover, the susceptibility for meropenem (10) and ertapenem (10) was also tested for *K. pneumoniae* isolates. The plates were incubated for 24 h at 37 °C. *E. coli* ATCC 25922 was used as a reference strain in susceptibility testing assays.

The screening of phenotypic ESBL production was carried out by the double-disk synergy test using cefotaxime, ceftazidime, and amoxicillin/clavulanic acid discs [39]. Isolates showing resistance to three or more antibiotic classes were considered as MDR.

### 2.3. DNA Extraction and Quantification

Genomic DNA from CTX/CAZ^R^ *E. coli* isolates was extracted using the boiled method [40], selecting three to five colonies in 1mL of sterile Milli-Q water for 8 min. Moreover, genomic DNA from *K. pneumoniae* isolates was extracted using the InstaGene Matrix (Bio-Rad).

### 2.4. Detection of Antibiotic Resistance Genes

*E. coli* and *K. pneumoniae* ESBL-positive isolates were screened by PCR/sequencing for the presence of the genes *bla*_CTX-M_ (different groups) [41], *bla*_SHV_ [42], *bla*_TEM_ [41], *bla*_CMY-2_, *bla*_DHA-1_, *bla*_KPC-2/3_, *bla*_VIM_, *bla*_VEB_, *bla*_OXA-48_, and *bla*_NDM_ [43,44]. The obtained amplicons were sequenced and analyzed by BLAST software, available at the National Center for Biotechnology Information [45]. The isolates were also screened for the presence of the genes encoding resistance for tetracycline (*tet*A, *tet*B) [46] and colistin (*mcr*-1) [47]. Moreover, the presence of the *int1* gene (encoding the integrase of class 1 integrons) was analyzed on the *E. coli* isolates obtained from blood origin [48]. Positive controls of the University of La Rioja were used in each of the PCRs carried out in this work.

### 2.5. Molecular Typing of Selected E. coli and K. pneumoniae Isolates

Phylogenetic classification of all 38 *E. coli* isolates was performed as previously reported by Clermont et al. [49], according to the existence of *arp*A, *chu*A, *yja*A, and TSPE4.C2 genes. The *E. coli* isolates of blood origin were further typed: (a) isolates of phylogroup B2 were screened for their affiliation to sequence type (ST) 131 by a specific PCR-region of the ST131 genome, as previously reported by Doumith et al. [50]; (b) the remaining blood isolates were typed by multilocus-sequence-typing (MLST) with seven housekeeping genes (*fum*C, *adk*, *pur*A, *icd*, *rec*A, *mdh*, and *gyr*B). The protocol described on PubMLST (Public databases for molecular typing and microbial genome diversity) was followed [51], and the allele combination was determined after sequencing of the seven genes to determine the sequence type (ST).

Moreover, the MLST was performed on selected *K. pneumoniae* isolates (based on the type of beta-lactamase genes they carried) by PCR/sequencing of seven housekeeping genes (*gap*A, *pho*E, *inf*B, *pgi*, *rpo*B, *ton*B, and *mdh*) as previously indicated [52].

## 3. Results

### 3.1. Antimicrobial Resistance Phenotype in E. coli and K. pneumoniae Isolates

Regarding the 38 CTX/CAZ^R^ *E. coli* isolates, all of them were ESBL-producers (Table 1). High rates of antibiotic resistance were observed among these isolates for ciprofloxacin (*n* = 36; 94.7%), tobramycin (*n* = 27; 71.1%), trimethoprim/sulfamethoxazole (*n* = 25; 65.8%), tetracycline (*n* = 22; 57.9%), gentamicin (*n* = 22; 57.9%) and amoxicillin + clavulanic acid (*n* = 21; 55.3%). Importantly, two isolates showed imipenem resistance (IMP^R^). All *E. coli* isolates were categorized as MDR. The detailed antimicrobial resistance profiles of these isolates are listed in Table 1.

All of the 24 CTX/CAZ^R^ *K. pneumoniae* isolates were ESBL producers (100%) and they were considered for further genetic resistance analysis. Interestingly, 16 ESBL-producing *K. pneumoniae* isolates were also resistant to ertapenem (66.7%), 17 isolates to imipenem (70.8%), and 14 to meropenem (58.3%) (Table 2). Beside for β-lactam antimicrobials, high resistance levels were recorded towards trimethoprim/sulfamethoxazole (*n* = 24; 100%), ciprofloxacin (*n* = 22; 91.6%), tetracycline (*n* = 18; 75%), gentamicin (*n* = 18; 75%), amoxicillin + clavulanic acid (*n* = 17; 70.8%) and cefoxitin (*n* = 7; 29.2%) (Table 2). Moreover, all of the *K. pneumoniae* isolates showed a MDR-phenotype.

### 3.2. Genetic Characteristics of ESBL- or Carbapenemase-Producing E. coli Isolates

Most of the 38 ESBL-producers *E. coli* isolates carried a *bla*_CTX-M_ gene, mainly the *bla*_CTX-M-15_ gene (*n* = 32; 84.2%) (Table 1). Furthermore, the *bla*_CTX-M-27_ gene was detected in one isolate (blood origin) and the *bla*_CTX-M-1_ was positive for another isolate (urine origin); the *bla*_CTX-M_ variant could not be detected in three additional isolates. Interestingly, two of the ESBL-positive isolates were also IMP^R^ and both of them carried the *bla*_KPC-2/3_ gene. Likewise, resistance to tetracycline was conferred, particularly, by the *tet*(A) (*n* = 16) or *tet*(B) (*n* = 3) genes. Moreover, the *mcr*-1 gene, conferring colistin resistance, was not detected among our clinical *E. coli* isolates. The *int*1 gene was found among most strains from blood origin (*n* = 12) (Table 1).

ESBL-positive *E. coli* isolates were ascribed mainly to the phylogenetic group B_2_ (*n* = 31; 75.6%), followed by A (*n* = 3), D (*n* = 2), and C (*n* = 1) phylogroups; according to Clermont et al. (2013) [34], the phylogenetic group associated with the remaining isolate was not conclusive. It is important to note that most of the ESBL-positive *E. coli* isolates from blood origin were typed as ST131/B2 (*n* = 18/20), although also we could identify two ST410/A isolates (Table 1).

### 3.3. Genetic Characteristics of ESBL-Producing K. pneumoniae Isolates

Regarding the 24 ESBL-producing *K. pneumoniae* isolates, the *bla*_CTX-M-15_ gene was the most common among these isolates (*n* = 16 isolates, 66.7%), which 11 of them belong to urine origin (Table 2). Furthermore, the *bla*_CTX-M-55_ gene was also found among these isolates (*n* = 2). A high diversity of SHV variants was detected, specifically SHV-27 (*n* = 8, ESBL- type), SHV-28 (*n* = 4), SHV-11 (*n* = 6, associated with CTX-M-15 or CTX-M-55), and SHV-12 (*n* = 6, ESBL-type and frequently associated with KPC2/3 gene) (Table 2). Regarding the *K. pneumoniae* isolates recovered from urine, 8 of them also carried the *bla*_TEM_ gene. Moreover, the *mcr*-1 gene was not detected among *K. pneumoniae* isolates.

Three sequence types (from 5 selected isolates) belonging to major international lineages of human pathogenic β-lactamases-producing *K. pneumoniae* isolates were identified as follows (sequence-type/associated ESBLs): ST15/CTX-M-15 + SHV-12, ST15/CTX-M-15 + SHV-28, ST280/CTX-M-15 + SHV-27, ST280/SHV-27, and ST147/SHV-12 (Table 2).

## 4. Discussion

In our study, the ESBL-producing *E. coli* isolates were mainly associated with the carriage of the *bla*_CTX-M-15_ gene (>80%) (Table 1). This finding is according to a previous result obtained in *E. coli* isolated from nosocomial settings in Portugal [19]. In Germany, this enzyme was also the most prevalent ESBL type (around 50% of the strains investigated) [17]. Recently, the *bla*_CTX-M-15_ gene was the most frequently detected among *E. coli* of both sick and healthy dogs and cats in Portugal [14,15]. Nevertheless, the *bla*_CTX-M-1_ was the most common ESBL gene among healthy students in Portugal (Lisbon), followed by the *bla*_CTX-M-15_ gene [13].

Other Portuguese reports showed the detection of qAmpC β-lactamase-producing *E. coli* recovered from clinical settings (DHA and/or CMY-2) [8] and non-clinical isolates (CMY-2) [20]. Furthermore, the CMY-2 encoding gene was recently reported among healthy/sick cats in Portugal [14]; although these genes were tested, they were not found among our isolates. The ESBL-positive *E. coli* isolates analyzed in this study were ascribed mainly to the phylogenetic group B2 (Table 1). These findings are according to the study conducted by Zhang et al. [53], showing the high prevalence of phylogenetic group B2 among urinary *E. coli* isolates from human patients in the USA. Similar results were recently obtained among blood isolates in Spain [54], as well as among healthy humans [55] and nursing home residents [22], both studies performed in Portugal. Furthermore, the B2 phylogroup was the most frequent among dogs with UTI in Portugal [56]. Contrastingly, other authors reported a phylogenetic diversity among clinical isolates from humans and/or pets in Germany [17] and Switzerland [11]. The B2 group is frequently associated with virulent extra-intestinal strains of humans/animals, frequently found in human ExPEC infections; the group D is also associated with extra-intestinal infections although at a lower rate. Contrastingly, B1 and A are ubiquitous and more associated with commensal *E. coli* both in humans and vertebrate animals.

The CTX-M-15 gene is frequently associated with a specific international lineage, the epidemic clone ST131, which has spread worldwide [15,57]. According to Belas et al. (2019) [56], the *E. coli* ST131/CTX-M-15/B2 is the most disseminated *E. coli* clonal group worldwide associated with an extensive antimicrobial resistance profile; this lineage was found among 90% of our *E. coli* clinical isolates (Table 1), which represent a public health concern. Particularly in Portuguese territory, ST131 clone was detected among residents of nursing homes [22], sick dogs [15], as well as in healthy humans [55].

Moreover, the ST410 clone was identified among *E. coli* isolates in this study (Table 1). This clone was previously reported in clinical dog samples in Liverpool [58], Switzerland [11], France [59], and Portugal [60]. According to a recent report, *E. coli* ST410 should be classified as a potential new high-risk international clone [25]. Regarding clinical isolates from humans, this clone is widely distributed, mainly in Danish patients [25] and those from Southeast Asia [61].

Considering the ESBL-producing *K. pneumoniae* isolates, the *bla*_CTX-M-15_ gene was the most commonly detected (66.7%) (Table 2), similarly with the results obtained in different countries in clinical samples from humans [3,5,10,62] and pets [63,64,65]. Moreover, this gene was the most commonly detected in *K. pneumoniae* isolated from septicemias obtained from the same hospital of the present study (CHTMAD, Vila Real, Portugal) [5]. This gene was also detected among non-hospitalized patients in Portugal, according to a recent study [66]. Furthermore, the *bla*_CTX-M-15_ gene was the most detected among companion animals in Italy [67] and Germany [17]. These data related with CTX-M-15 producers over-predominating is according with the majority of European countries, and this variant is widely distributed among both humans and pets.

Furthermore, a high diversity regarding SHV variants (ESBL and not ESBL ones) detected among our isolates is according to the results obtained on clinical isolates in Portugal (SHV-11/12), Spain (SHV-12), China (SHV-12/27), India (SHV-28), and Brazil (SHV-27) [4,34,68,69,70,71]. Moreover, Carvalho et al. (2021) [5] detected also different variants of the *bla*_SHV_ gene (*bla*_SHV-1_, *bla*_SHV-11_, or *bla*_SHV-27_) among *K. pneumoniae* isolates of blood cultures in the same hospital. It is important to note that a recent previous study showed the detection of *bla*_CTX-M-15_ gene (associated in most cases with *bla*_SHV-28_ gene) among *K. pneumoniae* isolated from healthy and sick dogs in Portugal [65]. Curiously, the *bla*_SHV-12_ (ESBL-variant) was widely reported in wildlife [31,72,73].

KPC-3-producing bacteria is endemic in many countries, but just recently appears in Portuguese hospitals. This fact is in line with our study and can be explained by the increase in carbapenems consumption. Specifically, according to the European Centre for Disease Prevention and Control (ECDC) [74], Portugal is considered one of the top carbapenem consumers in Europe (10.9% in 2019). The *bla*_KPC-2/3_ gene was also detected among Portuguese hospitalized patients [5,32,75], as well as in non-hospitalized patients [66]. According to Rodrigues et al. (2016) [66], the widespread distribution of KPC-3 among *K. pneumoniae* clinical isolates in Portugal was associated with successful high-risk clones ST147 and ST15, similarly with our results.

Three sequence types belonging to major international lineages of human pathogenic β-lactamases-producing *K. pneumoniae* were identified in this study. The ST15, associated with CTX-M-15, was detected in two isolates (Table 2). This clone was previously reported in other parts of the world among both hospital and community settings, namely in Portugal [4,5] and the Netherlands [76], indicating their global spread. Particularly, this clone was recently detected in a blood sample from the same hospital, associated with SHV-106/TEM production [5]. Furthermore, recent studies showed the presence of ST15/CTX-M-15 among healthy pets in Portugal [77], as well as sick dogs [65]. A possible explanation is the fact of HGT is caused by the proximity between humans and companion animals.

Curiously, the ST280 was found among two *K. pneumoniae* isolated from urine samples. This infrequent lineage was found among a patient in Korea [78] and in New York (in this last case, associated with the CTX-M-15 gene) [79].

On the other hand, the ST147 clone was associated with the spread of SHV-12 in a *K. pneumoniae* urine isolate in our study (Table 1); this lineage was also found among blood/urine samples in Portuguese hospitals, also associated with SHV-12 [12] or SHV-1/11+ KPC-2/3 [5].

## 5. Conclusions

ESBL-producing *Enterobacteriaceae* are endemic in different Portuguese clinical settings. In conclusion, the present study revealed the presence of ESBL-producing *Enterobacteriaceae* among clinical isolates from a hospital located in Northern Portugal, with the dominance of spread of CTX-M-15 co-harboring SHV-type ESBLs, and subsequently KPC-2/3 gene.

This work also showed the diversity of ESBLs associated with high-risk international clones (ST15, ST147, and ST280 clones in *K. pneumoniae*, and the ST131 and ST410 in *E. coli*), which play an important role in dissemination in hospital settings, and increased frequency in nosocomial infections with human health impact. A relentless vigilance of the evolution of the ESBL situation and the application of a One Health interdisciplinary approach is necessary to keep this problem under control.

## Figures and Tables

**Table 1:** Resistance phenotype and genotype in the 38 CTX/CAZ^R^ *Escherichia coli* isolates from clinical samples in a Portuguese hospital.

Sample	Origin	Date (Month Year)	Resistance Phenotype ^a^	ESBL Production ^b^	Β-lactamases	MLST ^c^	Resistant Genes/Integrons ^d^	PG ^e^
**X1068**	Blood	March 2017	AMC, CTX, CAZ, CIP, SXT	**P**	CTX-M-15, TEM	ST131	*int*1	B2
**X1080**	Blood	July 2018	AMC, CTX, CAZ, TET, TOB, CIP, GEN, SXT	**P**	CTX-M-15, TEM	ST131	*int*1, *tet*A	B2
**X1062**	Blood	December 2016	AMC, CTX, CAZ, TET, TOB, CIP, GEN, SXT	**P**	CTX-M-15	ST131	*int*1, *tet*A	B2
**X1063**	Blood	December 2016	AMC, CTX, CAZ, TET, TOB, CIP, GEN, SXT	**P**	CTX-M-15	ST131	*int*1, *tet*A	B2
**X1064**	Blood	December 2016	AMC, CTX, CAZ, TET, TOB, CIP, GEN, SXT	**P**	CTX-M-15	ST131	*int*1	B2
**X1065**	Blood	June 2017	AMC, CTX, CAZ, TET, TOB, CIP, GEN, SXT	**P**	CTX-M-15	ST131	*int*1, *tet*A	B2
**X1066**	Blood	June 2017	AMC, CTX, CAZ, TOB, CIP, GEN	**P**	CTX-M-15	ST131	ND	B2
**X1067**	Blood	February 2017	AMC, CTX, CAZ, TET, TOB, CIP, GEN, SXT	**P**	CTX-M-15	ST131	*int*1, *tet*A	B2
**X1069**	Blood	March 2017	AMC, CTX, CAZ, TOB, CIP, GEN	**P**	CTX-M-15	ST131	ND	B2
**X1070**	Blood	March 2017	AMC, CTX, CAZ, CIP, GEN	**P**	CTX-M-15	ST131	ND	B2
**X1071**	Blood	April 2017	AMC, CTX, CAZ, TET, TOB, CIP, SXT	**P**	CTX-M-15	ST131	*int*1, *tet*A	B2
**X1072**	Blood	April 2017	AMC, CTX, CAZ, TET, TOB, CIP, GEN, SXT	**P**	CTX-M-15	ST131	*int*1, *tet*A	B2
**X1073**	Blood	April 2017	AMC, CTX, CAZ, TET, TOB, CIP, GEN, SXT	**P**	CTX-M-15	ST131	*int*1, *tet*A	B2
**X1074**	Blood	April 2017	AMC, CTX, CAZ, TET, TOB, CIP, GEN, SXT	**P**	CTX-M-15	ST131	*int*1, *tet*A	B2
**X1075**	Blood	May 2017	AMC, CTX, CAZ, CIP	**P**	CTX-M-15	ST410	ND	A
**X1076**	Blood	May 2017	AMC, CTX, CAZ, TET, TOB, CIP	**P**	CTX-M-15	ST131	ND	B2
**X1105**	Blood	May 2017	AMC, CTX, CAZ, CIP	**P**	CTX-M-15	ST410	ND	A
**X1079**	Blood	July 2018	AMC, CTX, CAZ, TOB, CIP, GEN, SXT	**P**	CTX-M-15	ST131	*int*1	B2
**X1081**	Blood	August 2018	AMC, CTX, CAZ, TET, TOB, CIP	**P**	CTX-M-15	ST131	*tet*A	B2
**X1078**	Blood	July 2018	CTX, TET, CIP, SXT	**P**	CTX-M-27	ST131	ND	B2
**X3158**	Urine	February 2017	CTX, CAZ, TET, TOB, SXT, S	**P**	CTX-M-15	NT	ND	B2
**X3159**	Urine	February 2017	CTX, CAZ, TOB, CIP, GEN, S	**P**	CTX-M-15	NT	ND	B2
**X3160**	Urine	February 2017	CTX, CAZ, TET, TOB, CIP, GEN, S	**P**	CTX-M-15	NT	ND	B2
**X3161**	Urine	March 2017	CTX, CAZ, TOB, CIP, GEN, S	**P**	CTX-M-15	NT	ND	B2
**X3162**	Urine	April 2017	CTX, CAZ, TOB, CIP, GEN, S	**P**	CTX-M-15	NT	ND	D
**X3163**	Urine	May 2017	CTX, CAZ, TET, CIP, SXT, S	**P**	CTX-M-15	NT	*tet*A, *tet*B	D
**X3164**	Urine	May 2017	CTX, CAZ, CIP, SXT, S	**P**	CTX-M-15	NT	ND	B2
**X3165**	Urine	May 2017	CTX, CAZ, CIP, S	**P**	CTX-M-15	NT	ND	B2
**X3167**	Urine	June 2018	CTX, CAZ, TET, TOB, CIP, GEN, SXT, S	**P**	CTX-M-15	NT	*tet*A	B2
**X3168**	Urine	August 2018	CTX, CAZ, TET, TOB, CIP, GEN, SXT, S	**P**	CTX-M-15	NT	*tet*A	B2
**X3169**	Urine	August 2018	CTX, CAZ, TOB, CIP, GEN, SXT, S	**P**	CTX-M-15	NT	*tet*A	B2
**X3170**	Urine	August 2018	CTX, CAZ, TET, CIP, S	**P**	CTX-M-15	NT	*tet*B	B2
**X3173**	Urine	June 2018	AMC, FOX, CTX, CAZ, IMP, TET, CIP, SXT, S	**P**	CTX-M-15, KPC2/3	NT	*tet*B	A
**X3157**	Urine	February 2017	CTX, TET, SXT, S	**P**	CTX-M-1	NT	*tet*A	C
**X3155**	Urine	December 2016	CTX, TOB, CIP, SXT, S	**P**	CTX-M-variant	NT	ND	B2
**X3166**	Urine	May 2018	ERT, TOB, CIP, GEN, SXT, S	**P**	CTX-M-variant	NT	ND	B2
**X3171**	Urine	December 2016	CTX, TOB, CIP, SXT, S	**P**	CTX-M-variant	NT	ND	NC
**X3156**	Urine	June 2017	AMC, FOX, CTX, CAZ, IMP, TET, TOB, CIP, GEN, SXT, S	**P**	KPC2/3	NT	*tet*A	B2

**Legend:**^a^ AMC: amoxicillin + clavulanic acid; FOX: cefoxitin; CTX: cefotaxime; CAZ: ceftazidime; CHL: chloramphenicol; CIP: ciprofloxacin; TOB: tobramycin; GEN: gentamicin; SXT: trimethoprim + sulfamethoxazole; S: streptomycin; TET: tetracycline; IMP: imipenem; ^b^ P—Positive, N—Negative; ^c^ MLST—MultiLocus Sequence Typing; ^d^ NT: not tested; ND: not detected; NC: Not concluded; ^e^ Phylogroups.

**Table 2:** Resistance phenotype and genotype present in the 24 CTX/CAZ^R^ *Klebsiella pneumoniae* isolates from clinical samples in a Portuguese hospital.

Sample	Origin	Date (Month Year)	Antimicrobial Resistance Phenotype ^a^	ESBL Production ^b^	β-lactamases	MLST ^c^	Other Genes/Int ^d^
X2175	Urine	June 2017	AMC, CTX, CAZ, IMP, MRP, ERT, CIP, SXT, S	P	CTX-M-15, KPC-2/3, SHV-12, TEM	ST15	ND
X2143	Urine	December 2016	AMC, FOX, CTX, CAZ, IMP, MRP, ERT, TET, CIP, GEN, SXT, S	P	CTX-M-15, KPC-2/3, SHV-27, TEM	ST280	*tet*A
X2153	Urine	June 2017	AMC, FOX, CTX, CAZ, IMP, MRP, ERT, TET, CIP, GEN, SXT, S	P	CTX-M-15, KPC-2/3, SHV-27, TEM	NT	*tet*A
X2155	Urine	February 2017	AMC, CTX, CAZ, IMP, MRP, ERT, TET, CIP, GEN, SXT, S	P	CTX-M-15, KPC-2/3, SHV-27, TEM	NT	*tet*A
X2166	Urine	May 2017	AMC, CTX, CAZ, IMP, MRP, ERT, TET, CIP, GEN, SXT, S	P	CTX-M-15, KPC-2/3, SHV-27, TEM	NT	*tet*A
X3098	Urine	December 2016	CTX, CAZ, TET, CIP, TOB, GEN, SXT, S	P	CTX-M-15, SHV-27	NT	*tet*A
X3100	Urine	March 2017	CTX, CAZ, IMP, TET, CIP, TOB, SXT, S	P	CTX-M-15, SHV-27	NT	*tet*A
X3104	Urine	May 2017	AMC, CTX, CAZ, IMP, MRP, ERT, TET, CIP, TOB, SXT, S	P	CTX-M-15, SHV-11	NT	ND
X2157	Urine	April 2017	AMC, FOX, CTX, CAZ, IMP, MRP, ERT, CHF, CIP, GEN, SXT, S	P	CTX-M-15, KPC-2/3, SHV-28, TEM	**ST15**	ND
X3095	Urine	December 2016	CTX, CAZ, MRP, TET, CIP, TOB, GEN, SXT, S	P	CTX-M-15, SHV-28	NT	*tet*A
X3105	Urine	May 2017	AMC, CTX, CAZ, ERT, TET, CIP, TOB, GEN, SXT, S	P	CTX-M-15, SHV-28	NT	*tet*A
X3106	Urine	May 2017	CTX, CAZ, ERT, TET, CIP, TOB, GEN, SXT, S	P	CTX-M-55, SHV-11	NT	*tet*A
X2142	Urine	December 2016	AMC, FOX, CTX, CAZ, IMP, MRP, ERT, CHF, CIP, GEN, SXT, S	P	SHV-12, KPC-2/3, TEM	**ST147**	ND
X3092	Urine	December 2016	AMC, CTX, CAZ, IMP, TET, TOB, CIP, GEN, SXT, S	P	SHV-12	NT	ND
X3096	Urine	December 2016	CTX, CAZ, IMP, TET, CIP, SXT, S	P	SHV-12	NT	*tet*A
X3097	Urine	December 2016	CTX, CAZ, IMP, TET, TOB, CIP, GEN, SXT, S	P	SHV-12	NT	*tet*A
X3107	Urine	May 2017	AMC, FOX, ERT, CTX, CAZ, TET, CIP, SXT, S	P	SHV-12	NT	*tet*A
X2232	Urine	January 2017	AMC, CTX, CAZ, IMP, MRP, ERT, TET, CIP, GEN, SXT, S	P	SHV-27, KPC-2/3, TEM	**ST280**	*tet*A
X3085	Bronchial secretion	December 2017	AMC, FOX, CTX, CAZ, IMP, MRP, ERT, TOB, CIP, GEN, SXT, S	P	CTX-M-15, KPC-2/3, SHV-11	NT	ND
X3094	Pus	December 2017	CTX, CAZ, TET, TOB, GEN, SXT, S	P	CTX-M-15, SHV-11	NT	*tet*A
X3102	Biopsy	May 2017	AMC, CTX, CAZ, TOB, GEN, SXT, S	P	CTX-M-15, SHV-11	NT	ND
X3087	Bronchial secretion	December 2016	AMC, FOX, CTX, CAZ, IMP, MRP, ERT, TET, TOB, CIP, GEN, SXT, S	P	CTX-M-15, SHV-27	NT	*tet*A
X3088	Bronchial secretion	June 2017	AMC, CTX, CAZ, IMP, MRP, ERT, TOB, CIP, GEN, SXT, S	P	CTX-M-15, KPC-2/3, SHV-28	NT	ND
X3101	Catheter	April 2017	AMC, CTX, CAZ, IMP, MRP, ERT, TET, TOB, CIP, SXT, S	P	CTX-M-55, SHV-11	NT	*tet*A

**Legend:**^a^ AMC: amoxicillin + clavulanic acid; FOX: cefoxitin; CTX: cefotaxime; CAZ: ceftazidime; CHL: chloramphenicol; CIP: ciprofloxacin; TOB: tobramycin; GEN: gentamicin; SXT: trimethoprim + sulfamethoxazole; S: streptomycin; TET: tetracycline; IMP: imipenem; ERT: ertapenem; MRP: meropenem; **^b^** P—Positive, N—Negative; ^c^ MLST—MultiLocus Sequence Typing; ^d^ NT: not tested; ND: not detected.

## Data Availability

The data used to support the findings of this study are available from the corresponding author upon request.
